# On the Questionable Appeal of Glossy/Shiny Food Packaging

**DOI:** 10.3390/foods10050959

**Published:** 2021-04-28

**Authors:** Charles Spence

**Affiliations:** Department of Experimental Psychology, Anna Watts Building, University of Oxford, Oxford OX2 6BW, UK; charles.spence@psy.ox.ac.uk

**Keywords:** packaging, glossy, shiny, material properties, inner, outer, learned associations, evolutionary explanations

## Abstract

Those stimuli that have a shiny/glossy visual appearance are typically rated as both attractive and attention capturing. Indeed, for millennia, shiny precious metals and glossy lacquerware have been used to enhance the presentation, and thus the perception, of food and drink. As such, one might have expected that adding a shiny/glossy appearance/finish to the outer packaging of food and beverage products would also be desirable. However, the latest research appears to show that many consumers have internalised an association between glossy packaging and greasy (or unhealthy) food products, while matte packaging tends to be associated with those foods that are more natural instead. Furthermore, it turns out that many consumers do not necessarily appreciate the attempt to capture their attention that glossy packaging so often affords. At the same time, it is important to recognise that somewhat different associations may apply in the case of inner versus outer food and beverage packaging. Shiny metallic (inner) packaging may well prime (rightly or wrongly) concerns about sustainability amongst consumers. Given the research that has been published in recent years, food and beverage manufacturers/marketers should think very carefully about whether or not to introduce such shiny/glossy finishes to their packaging.

## 1. Introduction

For millennia, people have found shiny/glossy (the difference between glossy and shiny surfaces is that glossy is more the property of a surface, whereas shiny is more the property of reflecting an external light source) surfaces to be visually appealing in the context of food service (e.g., [1,2,3]; [4] p. 19), often transferring positive attributes to the experience of the food itself ([5] pp. 159–160; [6,7]). (As the Roman gourmand Apicius suggested in the first century AD: “an expensive silver platter would enhance the appearance of this dish materially” when referring to the plating of his Apician Dish (number 141; [8] p. 103).) What is more, Western cutlery made from shiny precious metals, such as gold and platinum has also held a certain appeal amongst the wealthy over the centuries ([9] p. 106; [10] p. 190). At the same time, it is worth noting that while aluminium foil was considered a futuristic material when it was first introduced back in the 1920s (e.g., by the Italian Futurists; see [11]), nowadays, like stainless steel, it has become an ubiquitous material, hence largely devoid of any symbolic meaning [12].

A shiny metallic appearance can help to set positively-valenced expectations [13] that then carry over to influence the tasting experience. For instance, metallic cups have been shown to evoke positive associations (i.e., of elegance), which can then give rise to enhanced tasting experiences [6,14,15,16]. At the same time, and as we have just seen, the symbolic meaning of different shiny metallic surfaces, such as that represented by aluminium foil, have undoubtedly changed over the decades ([11]; see also [17]). Furthermore, a good part of the appeal of glistening/glossy surfaces may traditionally have been linked to the dim indoor illumination (such as candlelight), as stressed by Tanizaki [3] in his extended aesthetics essay In Praise of Shadows. In the brightly-illuminated modern interior, however, such shiny precious metals can all too easily appear garish.

Going beyond the serviceware itself, at times, people have also been intrigued by the idea of eating foods that appear metallic (i.e., as when foods were occasionally covered in gold leaf for the lucky few who could afford it in Medieval England, see [4]) or iridescent (such as the surmullet fish in ancient Rome; see Andrews [18]). Many adverts for luxury items including alcoholic beverages use extrinsic attributes, such as gold and shininess, in order to try to convey prestige and style [19]. Note here also how there are likely to be cross-cultural differences in the meaning of food and beverage packaging appearance cues (such as shiny or glossy), as has previously been shown in the case of the meaning of packaging colour [20,21]. For instance, when used in outer packaging, a silver appearance is associated with the packaging of dairy products in the U.S., while being associated with fresh seafood in Norway [22,23]. Gold foil makes an occasional (both distinctive and eye-catching) appearance in the covering of the Lindt Goldhaser Easter chocolate bunny ([24] p. 84). That said, it has been argued that gold is not widely associated amongst customers with any particular product category or brand [25], excepting perhaps Nescafe’s Gold Blend instant coffee, Starbucks Gold Label, and the Lindt gold bunny. Meanwhile, giving a wine label a metallic visual appearance property can be used to effectively convey luxury/elegance [26], as has been successfully achieved in the world of heraldic-looking wine labels [27,28].

While shiny metallic and glossy surfaces/materials have long held an appeal to consumers, the question to be addressed in this review is whether the same is still true currently when it comes to the packaging of food and beverage products. However, before getting to that intriguing question, it is first worth summarising the literature concerning why exactly it should be that we find glossy and shiny surfaces attractive to begin with.

## 2. Why, Exactly, Are Glossy/Shiny Surfaces So Appealing?

Those stimuli that have a shiny/glossy visual appearance are typically rated as both attractive and attention capturing (e.g., [29,30,31]). Perhaps unsurprisingly, researchers have demonstrated that people prefer shiny to tarnished metal objects [32]. (According to the research, and contrary to European folklore, magpies are not attracted to shiny objects [33]). In the latter study, people were shown to prefer shiny silver coins over dull ones and mirror-polished copper cylinders over cylinders that had a brushed or dull surface. Glossy products are often associated with luxury [16]. According to one popular suggestion, humans are drawn to glossy surfaces as that may, evolutionarily speaking, have provided a robust cue as to the presence of fresh water, which would have been necessary for human survival (see [34,35,36,37] on visual cues to the perception of wetness). At the same time, it should also be noted that elsewhere in the literature, researchers have drawn attention to the association that exists between glossiness and freshness (e.g., of fish eyes, while glossy implying the wetness of the surface [38], not to mention fruits and vegetables [39,40]. Furthermore, in the context of foods, energy-dense fats often present a glossy visual appearance. Relevant here, a separate line of empirical research has demonstrated the attention-capturing properties of high-fat (i.e., energy dense) food images [41,42,43]. Hence, this might be taken to provide another alternative (non-exclusive) evolutionary explanation as to why glossy stimuli should be rated as so attractive (see also [44,45]).

People have been shown to find glossy shiny landscape photos more attractive than flat matte ones [46]. The researchers in the latter study manipulated the glossiness of paper on a between-participants basis while holding constant weight, colour quality, and resolution. One hundred participants viewed a number of landscape photos on either high-gloss photo paper or on identical paper where a flat, matte spray finish had been applied instead. A non-significant trend toward glossiness being rated as more attractive was observed. However, it turned out that in those individuals for whom the aesthetic appreciation characteristic of the ‘openness to experience’ trait (as assessed by means of a separate questionnaire; see [47]) was low, the glossy images were strongly preferred over the matte photos (thus replicating previous findings in the literature). By contrast, in those for whom aesthetic appreciation was high, there was no significant difference between image finishes.

There has been much research into the visual rendering of material qualities such as glossy [48,49,50,51,52,53,54], matte [55], metallic [12], translucent (e.g., [56,57]), shiny and iridescent (e.g., [30,58]). Furthermore, there is much interest in the digital rendering of such appealing visual appearance properties, based on the underlying image statistics that are related to specific perceptual judgments (e.g., see [59,60,61]; though see also [62]). There is also growing interest in understanding the neural mechanisms underpinning the various aspects of material perception (see [30] for a review).

How we interact with an object can also be influenced by whether or not it has been given a shiny/glossy surface appearance [16,63]. At the same time, physically touching surfaces/objects that are slippery has also been shown to enhance perceived gloss [64,65]. Meanwhile, Decré and Cloonan [66] have demonstrated that glossy (vs. matte) packaging influences the haptic perception of the roughness, thickness, and lightness of product packaging (see also [67,68]), which may, in turn, influence judgments of product quality/sophistication. In particular, products with a matte surface were rated as rougher, thicker, and heavier than those with a gloss surface (as assessed by three seven-point semantic differential scales).

One intriguing low-level stimulus feature that will be focused on here is glossiness: namely, whether objects appear relatively dull or shiny. The results of a handful of studies suggest that people prefer shiny materials, possibly because glossiness connotes water. In one early study, for instance, Coss and Moore [35] showed adults different papers varying in their surface finishes, such as glossy, flat, sandy, and sparkly papers. Semantic differential ratings revealed that the glossier papers were rated as “wetter” and as more appealing. Meanwhile, Meert et al. [36] presented images printed on glossy or plain paper to children and adults who had to rank-order the images from most to least attractive whilst also rating attractiveness. Once again, the glossy images were consistently rated as more attractive than the plain images.

## 3. What Is Wrong with Glossy/Shiny Packaging?

It has recently been argued that visual material perception represents something of a neglected area in the field of research on packaging design [40,44]. Indeed, the contrast in the amount of research is especially noticeable when compared with the vast literature that has been published over the years on the topic of packaging colour (see [69,70]; see also [26,71,72]). Beyond the hue of the product packaging, some researchers have also assessed the different meanings that consumers associate with light versus dark colours in the context of food products [73,74,75]. For example, Mead and Richerson [75] argued that healthier products tend to be associated with more highly saturated packaging colours.

One of the most intriguing (and popular) comparisons in the marketing literature on the material properties of packaging has been between matte versus glossy versions of the same packaging (e.g., [29,44,66,76,77]). The research shows that people are able to detect a glossy surface appearance very rapidly [78,79]. Given the evolutionary appeal of shiny/glossy surfaces, one might have expected that adding a glossy finish to outer packaging ought to be appealing to consumers (cf. [32,80,81,82]. Furthermore, given the positive sensation transference from metallic/glossy serviceware to food (as documented earlier), one might have expected that the same beneficial effect (i.e., sensation transference) would also occur in the case of the appearance properties of the (outer) packaging of food and drink (see [83,84], for the original suggestion that consumers’ feelings about product packaging may carry over to influence their feelings about the product itself). It turns out, however, that many contemporary consumers have developed a crossmodal association between glossy outer packaging and greasy unhealthy foods [77]. Consequently, the literature suggests that it is not necessarily always a good idea to give one’s outer packaging a glossy finish (at least not in the case of food and beverage products). In fact, a number of recent studies have highlighted how glossy packaging is nowadays associated (at least amongst the North American, Austrian, and Belgian participants who have been tested to date) with a number of negative attributes in the context of food and drink product packaging. Consumers appear to associate glossy packaging with those foods that are unnatural, unhealthy, and greasy [44,76,77], and with brands that are trying too hard to capture the attention of the consumer, thus lowering the perceived trustworthiness of the brand [29]. In other words, glossy packaging would often appear to carry negative connotations for consumers in the Food and Beverage (F&B) category currently. Confusing matters somewhat, though, other recent research has highlighted how, away from the context of food and drink (i.e., in the Home and Personal Care, HPC, categories), gloss packaging is associated with increased purchase intention and willingness to pay [66].

### 3.1. Outer Packaging

According to Han [29], North American consumers perceive glossy outer packaging to be too attention capturing. Han conducted four studies (thus far reported only as a brief conference paper) in which people’s impressions of a variety of product descriptions/packaging exemplars were compared in a matte versus gloss finish (see Table 1 for a summary of this and other studies that have compared matte vs. gloss product packaging). Han’s results revealed that across a range of food product categories, from granola to chocolate, matte packaging was preferred to gloss. In this case, part of the reason was simply that gloss packaging made it seem as though the company/brand was trying too hard to attract their customers’ attention, thus reducing the perceived trustworthiness of the brand. Han’s participants had to indicate which of the two versions of the product (matte or gloss) they would choose (on a seven-point scale) and also give a trustworthiness rating to the brand. The cereal described as being presented in matte packaging (in Experiment 1) was found to be preferred over the gloss package description, mediated entirely by perceived trustworthiness. This pattern of results was replicated in a second experiment now involving graphical representations of product packaging (of chocolate and granola). That said, Han [29], Experiment 3, went on to demonstrate that outside the context of food and drink, a glossy finish was deemed as being less unacceptable for a premium brand, such as Chanel, than it was for a generic (fake) brand. In other words, the tendency to consider glossy packaging to be less trustworthy was reduced for a high-quality or premium brand (at least outside the context of food and drink). In Han’s fourth experiment, the participants rated glossy packaging as more immediately attention capturing, whereas there was a borderline-significant suggestion for the participants to think that matte packaging held their attention for longer. (It would, however, be desirable to have objective measures of attentional capture to back-up these subjective ratings) Once again, glossiness led to lower levels of trustworthiness, based on attempts to capture their attention being seen as a marketing tactic by the participants.

It has been reported that matte packaging can help to increase the perceived naturalness of food products [76]. The latter researchers conducted a series of three studies in which their participants were shown product packaging of a range of everyday food products, including an unlabelled red plastic ketchup bottle (Experiment 1), raspberry soda and raspberry iced tea shown in labelled white bottles (Experiment 2a), and protein bar labels either with or without a natural product claim (Experiment 2b). The participants rated perceived naturalness, expected tastiness, and quality of the products on seven-point scales, as well as rating their purchase intent (Experiment 2). Experiments 1 and 2a were conducted in a mock-up Point-of-Sale in the lab in Austria (with multiple products), whereas Experiment 2b was conducted to imitate online product inspection showing only a single product at a time that the participant (USA MTurkers) could zoom in on. Experiment 1 also compared looking versus looking and handling the product packaging, though this factor did not influence the pattern of results obtained. The matte version of the packaging was rated as looking more natural when the product itself was considered fairly artificial (e.g., tomato ketchup, raspberry soda, and the protein bar without an explicit natural claim). The matte–natural association led on to follow-on effects (i.e., mediated the effects) on expected tastiness (all experiments) and increased willingness to purchase (Experiment 2).

Ye et al. [77] conducted a series of six studies showing firstly (based on audits of six North American retail stores) that unhealthy foods like potato chips tends to be come in glossy packaging whereas healthier snacks, like crackers, tend to be sold in matte packaging (Experiment 1). Of the 2656 packages classified, the majority of potato chips on shelf were in glossy packaging (76.8%) whereas only a minority of crackers were packaged in glossy packaging (4.5%). In Experiment 2, a new group of participants were asked explicitly about their expectations concerning matte and gloss packaging. As might have been expected, participants expected less healthy/more greasy snacks to come in glossy packaging. Thereafter, these researchers used a simplified version of the Implicit Association Test to demonstrate that North American consumers appear to have internalised this crossmodal association (or correspondence; Experiment 3). Experiment 4 demonstrated that those snack foods with glossy (matte) surfaces were inferred to be less (more) healthy. In Experiment 5, the researchers demonstrated that participants both poured less potato chips from a gloss bag than from a matte bag when instructed to serve 50 g (10.21 g vs. 11.77 g). Thereafter, they were also shown to consume slightly (but significantly) less when the potato chips came from a matte bag (Experiment 5). Finally, in Experiment 6, Ye et al. demonstrated that people were more likely to buy snack products (potato chips, corn chips, or popcorn) in glossy packaging from a burger truck (73% of sales) than from a salad truck (34% of sales being glossy). The suggestion was that those choosing to visit the burger truck were presumably focused more on taste than those who chose the salad truck—they were motivated to engage in tasty rather than healthful eating.

Ye et al. [77] argue for learned associations between unhealthy (i.e., greasy) foods and shiny packaging as the cause of the effects that they observed (so, in other words, the suggestion is that consumers internalise the natural statistic of the marketplace): namely, that greasy (and unhealthy) foods tend to be sold in glossy (rather than matte) packages. (It should be noted though that, at least in Spain, crisps (potato chips) that are sold in matte packaging tend to be more expensive than those sold in metallised glossy packaging [85].) However, as De Kerpel et al. [44] have pointed out subsequently, sugary products are often sold in glossy packaging, and consumers appear to have internalised the latter association too.

The influence of packaging glossiness on the perception of food products was recently investigated in a couple of studies reported by De Kerpel et al. [44]. These researchers first conducted an online study in which they probed participants about their expectations/beliefs concerning the taste qualities that they associated with food product packaging (for sweets and potato chips) having a glossy (vs. matte) finish. The results revealed that participants expected both products to come in glossy packaging. This questionnaire was then followed up by an in-store experiment in which shoppers at a tasting table in Belgium were invited to sample a new chocolate cranberry product from packaging that had either been given a glossy or a matte finish. De Kerpel et al. [44] also varied the packaging format (bag or can). Importantly, each participant rated only one of the four packaging formats, thus ensuring that undue attention was not focused on the finish of the packaging (i.e., matte vs. gloss). The participants were first asked about their expectations on handling the packaging. Seven-point semantic differential scales were used to assess expected fat content, expected sugar content of the new chocolate product. The participants were also asked about the expected healthiness, quality, and expensiveness of the product prior to tasting. The participants then got to sample the chocolates and rate their taste experience (tastes good, tastes appetising).

The results revealed that the expected fat content of the chocolates was higher, while the expected sugar content was lower, for the glossy as compared to the matte version of the packaging. Overall, matte packaging led to higher expected product health, expensiveness, and quality, while, at the same time, also enhancing rated taste experience on sampling the chocolates. Intriguingly, however, and in contrast to Marckhgott and Kamleitner [76], the finish of the packaging had no influence on the expected naturalness of the products, perhaps because the products were not perceived as artificial in the first place (though this is nothing more than a post hoc explanation.

De Kerpel et al.’s [44] research, like that of Ye et al. [77], therefore suggests that people associate glossy food packaging with those products having a higher fat content. An inference (or derived belief) that follows on from this is that a food product that is presented in a glossy package will be perceived as less healthy, less tasty, lower in quality, and cheaper. Thus, taken together, while the results of De Kerpel et al.’s [44] study demonstrate that Belgian consumers associate glossy product packaging with fat in food and with those products that are sugary, the glossy association with greasiness appears to dominate product perception, thus suggesting that the evolutionary account of many fats being glossy provides a better explanation for the data than simply learned associations [44,66,77] (There are, though, a number of interesting follow-up questions here concerning the differences, if any, between saturated and unsaturated fats (think avocado), and their visual associations (see also [44]).

At the same time, Decré and Cloonan [66] came to a somewhat different conclusion regarding people’s perception of glossy packaging based on a series of three between-participants experiments showing that manipulating packaging (glossy versus matte) not only influenced people’s perception of the haptic textural properties of packaging but also influenced their ratings of perceived product quality and attractiveness too. Giving the packaging of HPC products (shampoo, toothpaste, and face cream) a glossy finish led to increased ratings of product attractiveness, liking/beauty, and quality. This also had a knock-on effect on behavioural intentions, namely purchase intent but not on willingness to pay. It is striking how this is the only one of the five studies reviewed in Table 1 to find that glossy packaging enhanced product perception; it is also the only study to have been conducted entirely outside of the F&B space.

### 3.2. Limitations of the Research on Glossy vs. Matte Packaging Cues

One of the problems, or limitations, with those studies that have compared matte versus gloss packaging is that the two packaging formats tend to be assessed in isolation. Furthermore, there is also a concern that having participants directly compare matte versus gloss versions of the same packaging may draw the participants’ attention to this particular aspect of the visual appearance (since it is the only one that changes) in a way that is normally not be the case (see [46] for a similar criticism levelled at those studies that have manipulated whether pictures have a glossy vs. matte surface). Important in this regard, therefore, De Kerpel et al. [44], Experiment 2, conducted one of the only between-participants studies of gloss versus matte packaging in the ecologically-valid context of a tasting table in a store (meaning that each participant only experienced one example of product packaging). That said, Decré and Cloonan’s [27] studies in the HPC category were also conducted on a between-participants basis. This is obviously going to be more realistic given that in the marketplace, most products are typically only displayed in one finish (i.e., either matte or gloss).

Most commercial food and beverage products need to compete for the consumer’s visual attention in the food aisle (see also [44], who highlight much the same point), and increasingly online too [86,87,88,89]. As such, one might want to question just how informative the findings of those studies in which packaging exemplars are presented in isolation actually are (cf. [25]). It would certainly be interesting in future research to investigate whether glossy objects (specifically food and beverage packages) pop-out from realistic shelves of products in the context of visual search [90,91,92,93].

### 3.3. Inner Packaging

The appearance properties of the outer packaging of food and beverage products are normally only one element in the consumers’ total packaging experience. The visual material properties of inner packaging materials are potentially also important too [70,94,95]. Relevant here, in his history of consumer food packaging in the West (in truth, primarily North America), Hine has highlighted the longstanding appeal and incorporation of shiny metallic elements in the inner packaging of many food and beverage products [96]. Indeed, Nestlé were criticised in some quarters back in 2001 when they switched their iconic foil inner sleeve (and paper outer) for their KitKat after 65 years for a single flow-wrap plastic foil packaging [97]. Part of the reason for the change may have been growing concerns about the sustainability of different packaging formats, given that nowadays metallic packaging is not universally popular, due to concerns about sustainability, etc. [70,98].

There is a growing recognition that the visual appearance of inner packaging, and even the appearance of the product against inner surface of the inner packaging, e.g., as when consuming crisps (potato chips) direct from the bag, can (and should) be considered as an important part of the total multisensory product experience. It may be that a glossy or coloured inner packaging is interpreted rather differently by the consumer than exactly the same visual appearance property when displayed as outer packaging [99]. That said, there is undoubtedly a shortage of research on the impact of inner packaging colour/finish which, note, is likely to be especially important for those food products that are normally consumed direct from the packaging, such as is often the case for crisps/potato chips [100]. The main point to stress here is that when considering packaging recommendations concerning the use of matte versus gloss packaging, the answer may depend on whether one is considering outer vs. inner packaging.

## 4. Conclusions

As this review of the literature has hopefully made clear, adding a glossy/shiny finish to one’s packaging (be it outer or inner product packaging) is not always a good idea in terms of managing/enhancing the consumer’s expectations/associations [101]. In part, this is because of growing concerns about sustainability in the case of metallic inner packaging [98,102]. However, researchers have also highlighted the association that has been internalised by consumers that greasy foods tend to come in glossy packaging [77]. According to Han [29], adding gloss to one’s packaging can make it seem like a brand (especially one that is not especially well established) is trying too hard to capture the consumers’ attention, and for those aiming to convey the notion of a natural product, matte packaging seems better [76]. De Kerpel et al. [44] have argued for an evolutionary account linking glossiness with fattiness, rather than the learned associations account, given that consumers have also internalised the association between sugary products and glossy packaging.

At the same time, it is important to stress the fact that the majority of research in this area has been conducted in a small number of Western markets (see Table 1). This obviously leaves open the possibility that consumers in other parts of the world may have internalised somewhat different associations/conventions with such unbranded visual appearance cues as represented by gloss/matte packaging. Indeed, given that cross-cultural differences in the meaning of packaging colour have been well documented, it would perhaps be surprising were the same not also found to be true of metallic, shiny/glossy finishes on packaging. Furthermore, looking to the future, it might be beneficial to adopt more implicit experimental approaches to the study of packaging design in the case of food and beverage products, rather than those that have relied on explicit subjective judgments ([103,104], for a couple of implicit testing paradigms that would be appropriate to assess the associations and appeal of food and beverage packaging; and see Ye et al. [77], Experiment 3).

Finally, when considering the meaning of visual appearance cues in the case of food and beverage packaging, it is important to stress the important differences that may sometimes be present when the same packaging material is incorporated into inner versus outer packaging. That said, to date, the majority of the research has tended to focus on the different association/meaning that might be associated with outer packaging. There may also be some interesting/salient individual differences to be aware of in this space too (cf. [105,106,107]).

It will also be interesting in future research to examine whether the meaning/associations of a glossy versus matter surface finish might differ depending on the product contained within. It would be interesting to investigate whether a glossy finish might prime associations with fat in greasy products such as potato chips, while perhaps connoting freshness in the case of the packaging of seafood (cf. [38,77]). Additionally, should a glossy finish be added to the packaging of bottled (or nowadays canned) water then perhaps the link between glossy and the presence of water will be preferentially primed instead. Were such a suggestion to be validated by future research, then it might mean that there are no absolute associations with glossy packaging. Rather, the meaning is constrained both by the category of (food) product and perhaps by the country in which the product is sold. The same is presumably also true in the case of shiny metallic packaging features as well (cf. [108]).

In future research, it will be important to look at actual sales, rather than just willingness to pay (cf. [44,109]). Note here only how Ye et al. [77], Experiment 6, is the only study published to date that has actually assessed sales of matte vs. gloss packaging. Ultimately, based on the available research, the benefit of matte over gloss finish would appear to be reduced for those foods that are already perceived as natural [76], and possibly also for those brands that are perceived as premium [29]. Meanwhile, outside the F&B category (i.e., in HPC), different rules may apply [66]. As Ye et al. [77] suggest, it would also be interesting to know whether automobiles with a shinier paint finish are also perceived as faster, and more luxurious [110].

## Figures and Tables

**Table 1:** Summary of studies of glossy vs. matte outer product packaging.

Study (& Where Conducted)	Experiment/No. of Participants	Stimuli Compared	Preferred Packaging	Comment
Han (2018) (USA) [29]	E1 (121)	Written description of cereal package	Matte	Glossy packaging rated as less trustworthy. Difference attenuated for prestige brand. Glossy packaging more likely to capture attention.
	E2 (121)	Chocolate & granola	Matte	
	E3 (234)	Prestige (Chanel) vs. generic brand	Matte	
	E4 (252)	Glossy vs. matte packaging	Matte	
Decré & Cloonan (2019) (French, MTurk, USA) [66]	E1 (43)	Shampoo	Gloss	Glossy finish affects expected haptic properties. Higher purchase intent for glossy, rated as higher quality & more attractive
	E2 (92)	Toothpaste	Gloss	
	E3 (95)	Face cream	Gloss	
Marckhgott &Kamleitner (2019)(Austria, Austria,& USA MTurk) [76]	E1 (136)	Bottle of ketchup	Matte	Matte packaging rated as more natural for those products perceived as artificial, leading to increased expected tastiness & willingness to buy (E2).
	E2a (240)	Raspberry soda & raspberry iced tea shown in white bottles	Matte	
	E2b (231)	Protein bars w/without natural claim	Matte	
Ye et al. (2019) (Mturkers, USA) [77]	E4 (156)	Potato chips	Matte	Matte snack packaging perceived as healthier (E4), leading people to consume more (E5), & more likely to by snack in glossy package from burger truck than from salad truck
	E5 (203)	Potato chips	Matte	
	E6 (sales)	18 glossy & 18 matte snack packages	Matte	
De Kerpel et al. (2020) (Belgium) [44]	E1 (184)	Online study of sweets and potato chips	Matte	with both high fat & sugarGlossy packaging associatedreduced taste, naturalness, etc.
	E2 (178)	New chocolate-covered cranberry product	Matte	
